# Does aortic calcification really affect anastomotic leakage after rectal cancer surgery?

**DOI:** 10.1097/MD.0000000000038860

**Published:** 2024-07-12

**Authors:** Yu-Hang Diao, Jian Chen, Yang Liu, Dong Peng, Dong Yang

**Affiliations:** a Department of Gastrointestinal Surgery, The First Affiliated Hospital of Chongqing Medical University, Chongqing, China; b Department of Radiology, Qijiang People’s Hospital, Chongqing, China; c Department of Radiology, The First Affiliated Hospital of Chongqing Medical University, Chongqing, China.

**Keywords:** anastomotic leakage, calcification, computed tomography, rectal cancer, surgery

## Abstract

The purpose of the current study was to analyze whether aortic calcification had impact on the anastomotic leakage (AL) after rectal cancer (RC) surgery. We collected patients’ information from January 2011 to January 2020 in a single teaching hospital. Preoperative computed tomography images were obtained. Abdominal aortic calcification (AAC), superior mesenteric aortic calcification, and inferior mesenteric aortic calcification were recorded. The difference of AL and grade C AL was calculated. A total of 2412 RC patients were included in this study. Ninety-seven (4.0%) RC patients experienced AL and 47 (1.9%) RC patients experienced grade C AL. The amount of AAC, superior mesenteric aortic calcification, and inferior mesenteric aortic calcification was 1546 (64.1%), 128 (5.3%), and 31 (1.3%). The AL group had higher portion of AAC (*P* = .019) than the no AL group, and the grade C AL group had higher portion of AAC (*P* = .016) than the no grade C AL group. In univariate logistic regression analysis, AAC was a significant potential factor for AL (*P* = .021, OR = 1.739, 95% CI = 1.088–2.779) and grade C AL (*P* = .019, OR = 2.339, 95% CI = 1.115–4.986). However, in multivariate logistic regression, AAC was not an independent predictive factor for AL (*P* = .157, OR = 1.443, 95% CI = 0.871–2.358) or grade C AL (*P* = .064, OR = 2.055, 95% CI = 0.960–4.399). AAC was associated with higher amount of AL and grade C AL, however, AAC was not an independent predictive factor for AL or grade C AL.

## 1. Introduction

Colorectal cancer (CRC) is the third most common malignancy in the world and the second leading cause of mortality.^[1,2]^ Among the various treatment methods, surgery is the main milestone.^[3,4]^ Rectal cancer (RC) accounts for >30% of CRC cases,^[5]^ and total mesorectal excision is the standard of care.^[6]^

Anastomotic leakage (AL) is a common severe complication after RC surgery, with an incidence approximately ranging from 10% to 20%.^[7–13]^ The occurrence of AL significantly increases the length of hospital stay and the risk of postoperative mortality.^[14,15]^ Furthermore, AL also increases the risk of postoperative recurrence and reduces the overall survival of RC patients.^[16,17]^

A meta-analysis that included 4 studies reported that abdominal vascular calcification was associated with increased risk of AL after colorectal surgery,^[18]^ however, the included patients were only 496 patients (rectal cancer, colon cancer patients or benign colorectal diseases). It is hard to get accurate results from these studies with a small amount of data. Moreover, rectal cancer had higher portion of AL than colon cancer,^[19]^ which might provide more significance for analysis. Therefore, the purpose of the current study was to analyze whether aortic calcification had impact on AL after RC surgery.

## 2. Materials and methods

### 2.1. Patients

The patients’ information was retrospectively collected from January 2011 to January 2020 in a single teaching hospital. This study was conducted in accordance with the World Medical Association Declaration of Helsinki. Ethical approval from the institutional review board of the First Affiliated Hospital of Chongqing Medical University was obtained (2022-132-1) and all patients signed informed consents.

### 2.2. Inclusion and exclusion criteria

RC patients (n = 3235) who underwent radical surgery were included in a single teaching hospital. The exclusion criteria were as follows: 1. non-R0 RC surgery (n = 25); 2. recurrent RC surgery (n = 13); 3. incomplete medical records (n = 77); 4. Hartmann or Miles procedure (n = 650); and 5. incomplete preoperative computed tomography (CT) images (n = 58). Finally, a total of 2412 patients with complete preoperative CT images were included in the current study.

### 2.3. Image acquisition

All examinations were performed with 16- or 64-section CT scanners in our hospital and met the following minimum standard: 1. contrast material- enhanced abdominal and pelvic preoperative CT protocols were performed in one examination procedure; 2. all examination contained at least plain scan, arterial phase, venous phase, and at least one of arterial or venous phase which was reconstructed with a thin slice thickness ranging from 0.625 to 1.0 mm; 3. an iodinated contrast material bolus with a saline solution chaser was administered intravenously in all patients; 4. a region of interest was placed in the abdominal aorta, and image acquisition was automatically initiated once a selected threshold was reached within this region of interest with bolus tracking.

### 2.4. Image evaluation

When patients with more than one preoperative CT examination, only the first diagnostic examination was evaluated. All preoperative CT were reviewed by radiologist one (with 8 years of experience in gastrointestinal radiology) and cross-checked by radiologist 2 (with 15 years of experience in gastrointestinal radiology). In cases with disagreements, consensus was reached after discussion. Both readers were blinded to patient- and operation-related characteristics and clinical outcomes in terms of AL. All evaluations were conducted on multiple planar reconstruction-based transverse, coronal, and sagittal images.

### 2.5. Definition

**Figure 1.**
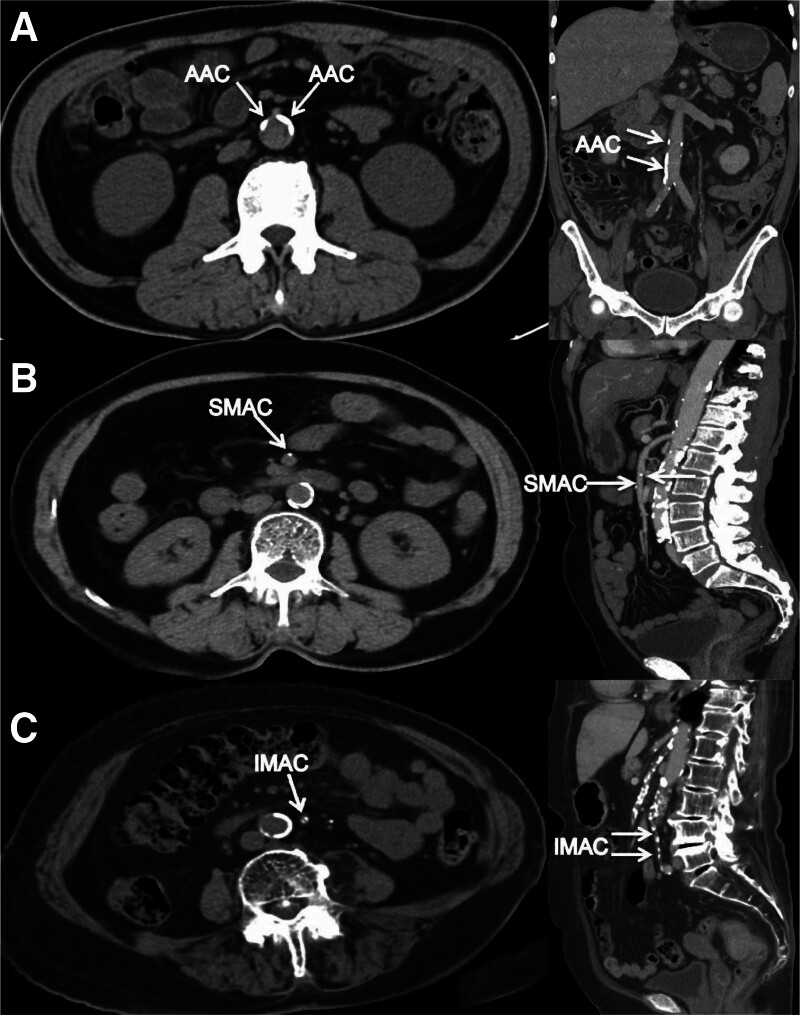
Examples of calcification on preoperative CT images in RC patients. (A) Image showing the AAC; (B) image showing the SMAC; (C) image showing the IMAC. AAC = abdominal aortic calcification; CT = computed tomography; IMAC = inferior mesenteric aortic calcification; RC = rectal cancer; SMAC = superior mesenteric aortic calcification.

Abdominal aortic was defined as the range from superior mesenteric artery (SMA) level to the arteria iliaca communis level. We collected the calcification of 3 sites: abdominal aortic calcification (AAC), superior mesenteric aortic calcification (SMAC), and inferior mesenteric aortic calcification (IMAC). AAC was defined as the range from SMA level to the arteria iliaca communis level; SMAC was defined as the calcification in the SMA; IMAC was defined as the calcification in the inferior mesenteric artery. The CT images of AAC, SMAC, and IMAC were shown in Figure 1.

The tumor node metastasis stage was diagnosed according to the AJCC 8th Edition and standard rectal cancer surgery was conducted (total or tumor-specific mesorectal excision with D3 lymphadenectomy was performed).^[20]^ Tumor location was recorded. The tumor location was defined as low, mid, and upper location. The low location was defined as <5 cm to the anal margin; the mid location was defined as 5 to 10 cm to the anal margin; The upper location was defined as >10 cm to the anal margin.

AL was defined as the discontinuation of a communication between the intra- and extraluminal compartments arising from a defect in the intestinal wall at the anastomotic site 30 days after RC surgery.^[21]^ The degree of AL was divided into 3 groups: grade A, grade B, and grade C. Grade A AL required no active intervention; grade B AL required active intervention but not relaparotomy; grade C AL required relaparotomy.^[15]^

### 2.6. Medical data collection

We collected medical data through inpatient system and CT data through picture archiving and communication image systems. The medical data included age, sex, body mass index, patients’ habits (smoking and drinking), hypertension, type 2 diabetes mellitus (T2DM), surgical methods, tumor size, tumor location, tumor node metastasis stage, and AL occurrence. The CT data included AAC, SMAC, and IMAC.

### 2.7. Statistical analysis

Continuous variables are expressed as the mean ± SD, and independent-sample t test is used to compare the difference between the AL group and the no AL group, and the grade C AL group with the no grade C AL group. Frequency variables are expressed as n (%), and Chi-square test or Fisher exact test is used. Univariate logistic regression analysis was conducted to find potential predictors for AL and grade C AL. Factors (when the *P* value was <.05) were included in the multivariate logistic regression to identify the independent predictors for AL and grade C AL. Data were analyzed using SPSS (version 22.0) statistical software. A bilateral *P* value of <.05 was considered statistically significant.

## 3. Results

### 3.1. Patients

**Figure 2.**
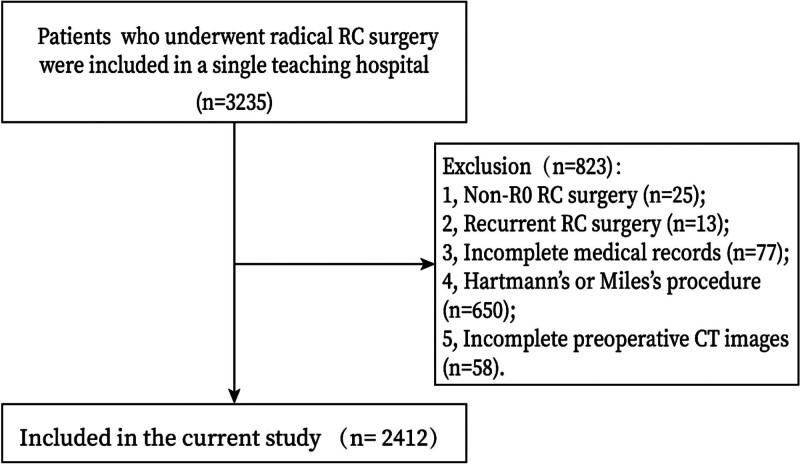
Flow chart of patient selection. CT = computed tomography, RC = rectal cancer.

A total of 2412 RC patients were included in this study according to the inclusion and exclusion criteria. The flow chart of patient selection was shown in Figure 2. There were 1506 (62.4%) males and 906 (37.6%) females. The amount of AAC, SMAC, and IMAC was 1546 (64.1%), 128 (5.3%), and 31 (1.3%). Ninety-seven (4.0%) RC patients experienced AL and 47 (1.9%) RC patients experienced grade C AL. Other baseline information was shown in Table 1.

**Table 1:** Clinical characteristics of RC patients.

Characteristics	No. 2412
Age, year	62.0 ± 11.8
Sex Male Female	1506 (62.4%) 906 (37.6%)
BMI, kg/m^2^	22.6 ± 3.2
Smoking	942 (39.1%)
Drinking	761 (31.6%)
Hypertension	560 (23.2%)
T2DM	243 (10.1%)
Laparoscopy	2245 (93.1%)
AAC	1546 (64.1%)
SMAC	128 (5.3%)
IMAC	31 (1.3%)
Tumor size	
<5 cm	1642 (68.1%)
≥5 cm	770 (31.9%)
Preventive ileostomy	329 (13.6%)
Neoadjuvant therapy	193 (8.0%)
Tumor location	
Low	928 (38.5%)
Middle-upper	1484 (61.5%)
TNM stage	
I	619 (25.7%)
II	867 (35.9%)
III	926 (38.4%)
AL	97 (4.0%)
Grade C AL	47 (1.9%)

Variables are expressed as the mean ± SD, n (%).

AAC = abdominal aortic calcification, AL = anastomotic leakage, BMI = body mass index, IMAC = inferior mesenteric aortic calcification, RC = rectal cancer, SMAC = superior mesenteric aortic calcification, T2DM = type 2 diabetes mellitus.

### 3.2. Comparison between AL and no AL

There were 97 patients in the AL group and 2315 patients in the no AL group. We compared the difference between the AL group and the no AL group and found that the AL group had higher portion of males (*P* < .01), smoking (*P* = .001), drinking (*P* = .006), hypertension (*P* = .037), T2DM (*P* = .032), lower tumor location (*P* < .01), and AAC (*P* = .019). There was no significant difference in SMAC (*P* = .596) or IMAC (*P* = 1.000) (Table 2).

**Table 2:** Comparison between AL and no AL.

Characteristics	AL (97)	No AL (2315)	*P* value
Age, year	61.2 ± 10.9	62.0 ± 11.8	.505
Sex Male Female	77 (79.4%) 20 (20.6%)	1429 (61.7%) 886 (38.3%)	<.01*
BMI, kg/m^2^	23.0 ± 3.4	22.6 ± 3.2	.213
Smoking	53 (54.6%)	889 (38.4%)	.001*
Drinking	43 (44.3%)	718 (31.0%)	.006*
Hypertension	31 (32.0%)	529 (22.9%)	.037*
T2DM	16 (16.5%)	229 (9.9%)	.032*
Laparoscopy	90 (92.8%)	2155 (93.1%)	.908
AAC	73 (75.3%)	1473 (63.6%)	.019*
SMAC	4 (4.1%)	124 (5.4%)	.596
IMAC	1 (1.0%)	30 (1.3%)	1.000
Tumor size			.500
<5 cm	63 (64.9%)	1579 (68.2%)	
≥5 cm	34 (35.1%)	736 (31.8%)	
Preventive ileostomy	17 (17.5%)	312 (13.5%)	.255
Neoadjuvant therapy	8 (8.2%)	185 (8.0%)	.927
Tumor location			<.01*
Low	63 (64.9%)	865 (37.4%)	
Middle-upper	34 (35.1%)	1450 (62.6%)	
TNM stage			.120
I	31 (32.0%)	588 (25.4%)	
II	38 (39.2%)	829 (35.8%)	
III	28 (28.8%)	898 (38.8%)	

AAC = abdominal aortic calcification, AL = anastomotic Leakage, BMI = body mass index, IMAA = inferior mesenteric aortic calcification, RC = rectal cancer, SMAC = superior mesenteric aortic calcification, T2DM = type 2 diabetes mellitus, TNM = tumor node metastasis.

* Variables are expressed as the mean ± SD, n (%), *P*-value <.05.

### 3.3. Comparison between grade C AL and no grade C AL

There were 47 RC patients who experienced grade C AL, we compared the grade C AL group with the no grade C AL group. The grade C AL group had higher portion of males (*P* = .043), smoking (*P* = .001), hypertension (*P* = .034), lower tumor location (*P* < .01), and AAC (*P* = .016). No significant difference was found in SMAC (*P* = 1.000) or IMAC (*P* = 1.000) (Table 3).

**Table 3:** Comparison between grade C AL and no grade C AL.

Characteristics	Grade C AL (47)	No grade C AL (2365)	*P* value
Age, year	62.0 ± 11.5	62.0 ± 11.8	.970
Sex Male Female	36 (76.6%) 11 (23.4%)	1470 (62.2%) 895 (37.8%)	.043*
BMI, kg/m^2^	23.0 ± 3.3	22.6 ± 3.2	.444
Smoking	29 (61.7%)	913 (38.6%)	.001*
Drinking	21 (44.7%)	740 (31.3%)	.050
Hypertension	17 (36.2%)	543 (23.0%)	.034*
T2DM	8 (17.0%)	235 (9.9%)	.135
Laparoscopy	44 (93.6%)	2201 (93.1%)	1.000
AAC	38 (80.9%)	1508 (63.8%)	.016*
SMAC	2 (4.3%)	126 (5.3%)	1.000
IMAC	0 (0.0%)	31 (1.3%)	1.000
Tumor size			.751
<5 cm	33 (70.2%)	1609 (68.0%)	
≥5 cm	14 (29.8%)	756 (32.0%)	
Preventive ileostomy	4 (8.5%)	325 (13.7%)	.301
Neoadjuvant therapy	1 (2.1%)	192 (8.1%)	.175
Tumor location			<.01*
Low	39 (83.0%)	889 (37.6%)	
Middle-upper	8 (17.0%)	1476 (62.4%)	
TNM stage			.414
I	16 (34.0%)	603 (25.5%)	
II	15 (32.0%)	852 (36.0%)	
III	16 (34.0%)	910 (38.5%)	

AAC = abdominal aortic calcification, AL = anastomotic leakage, BMI = body mass index, CHD = coronary heart disease, IMAC = inferior mesenteric aortic calcification, RC = rectal cancer, SMAC = superior mesenteric aortic calcification, T2DM = type 2 diabetes mellitus, TNM = tumor node metastasis.

* Variables are expressed as the mean ± SD, n (%), *P*-value <.05.

### 3.4. Univariate and multivariate logistic regression analysis of AL

The AAC was significantly higher in the AL group and grade C AL group, therefore, to further analyze whether AAC was a predictive factor of AL/ grade C AL, univariate and multivariate logistic regression analysis were conducted.

In univariate logistic regression analysis, AAC was a significant potential factor for AL (*P* = .021, OR = 1.739, 95% CI = 1.088–2.779) and grade C AL (*P* = .019, OR = 2.339, 95% CI = 1.115–4.986). However, in multivariate logistic regression, AAC was not an independent predictive factor for AL (*P* = .157, OR = 1.443, 95% CI = 0.871–2.358) or grade C AL (*P* = .064, OR = 2.055, 95% CI = 0.960–4.399) (Tables 4 and 5).

**Table 4:** Univariate and multivariate logistic regression analysis of AL.

Risk factors	Univariate analysis		Multivariate analysis	
	OR (95% CI)	*P* value	OR (95% CI)	*P* value
Age, year	0.994 (0.977–1.011)	.504		
Surgical methods (open/laparoscopic)	1.048 (0.478–2.298)	.908		
Sex (male/female)	2.387 (1.449–3.937)	.001*	1.919 (1.044–3.534)	.036*
BMI, Kg/m^2^	1.041 (0.977–1.109)	.213		
Hypertension (yes/no)	1.586 (1.024–2.456)	.039*	1.443 (0.904–2.304)	.125
T2DM (yes/no)	1.817 (1.045–3.160)	.034*	1.662 (0.918–3.007)	.093
Tumor location (low/middle-upper)	3.106 (2.030–4.753)	<.01*	3.155 (2.051–4.853)	<.01*
Neoadjuvant therapy (yes/no)	1.035 (0.494–2.167)	.927		
Tumor stage (III/II/I)	0.770 (0.597–0.994)	.045*	0.775 (0.598–1.006)	.056
Smoking (yes/no)	1.932 (1.284–2.907)	.002*	1.227 (0.703–2.139)	.471
Drinking (yes/no)	1.771 (1.175–2.669)	.006*	1.167 (0.695–1.959)	.560
Tumor size (≥5/<5), cm	1.158 (0.756–1.773)	.500		
AAC	1.739 (1.088–2.779)	.021*	1.443 (0.871–2.358)	.157
SMAC	0.760 (0.275–2.101)	.597		
IMAC	0.793 (0.107–5.879)	.821		

AAC = abdominal aortic calcification, AL = anastomotic leakage, BMI = body mass index, CI = confidence interval, IMAA = inferior mesenteric aortic calcification, OR = odds ratio, SMAC = superior mesenteric aortic calcification, T2DM = type 2 diabetes mellitus.

* *P*-value <.05.

**Table 5:** Univariate and multivariate logistic regression analysis of grade C AL.

Risk factors	Univariate analysis		Multivariate analysis	
	OR (95% CI)	*P* value	OR (95% CI)	*P* value
Age, year	1.000 (0.976–1.025)	.970		
Surgical methods (open/laparoscopic)	0.915 (0.281–0.979)	.883		
Sex (male/female)	1.992 (1.009–3.937)	.047*	1.047 (0.421–2.604)	.921
BMI, kg/m^2^	1.036 (0.947–1.133)	.444		
Hypertension (yes/no)	1.901 (1.041–3.474)	.037*	1.860 (0.993–3.481)	.052
T2DM (yes/no)	1.859 (0.859–4.026)	.116		
Tumor location (low/middle-upper)	8.094 (3.765–17.398)	<.01*	8.487 (3.935–18.304)	<.01*
Neoadjuvant therapy (yes/no)	0.246 (0.034–1.794)	.167		
Tumor stage (III/II/I)	0.814 (0.567–1.170)	.266		
Smoking (yes/no)	2.526 (1.415–4.640)	.002*	2.219 (0.997–4.940)	.051
Drinking (yes/no)	1.774 (0.991–3.173)	.053		
Tumor size (≥5/<5), cm	0.903 (0.480–1.697)	.751		
AAC	2.399 (1.155–4.986)	.019*	2.055 (0.960–4.399)	.064
SMAC	0.790 (0.189–3.293)	.746		
IMAC	0.000 (0.000-/)	.998		

AAC = abdominal aortic calcification, AL = anastomotic leakage, BMI = body mass index, CHD = coronary heart disease, CI = confidence interval, IMAA = inferior mesenteric aortic calcification, OR = Odds ratio, SMAC = superior mesenteric aortic calcification, T2DM = type 2 diabetes mellitus.

* *P*-value <.05.

In terms of AL, males (*P* = .036, OR = 1.919, 95% CI = 1.044–3.534) and lower tumor location (*P* < .01, OR = 3.155, 95% CI = 2.051–4.853) were independent predictive factors. Furthermore, lower tumor location (*P* < .01, OR = 8.487, 95% CI = 3.935–18.304) were independent predictive factors for grade C AL (Tables 4 and 5).

## 4. Discussion

A total of 2412 RC patients were included in this study. The AL group had higher portion of AAC than the no AL group, and the grade C AL group had higher portion of AAC than the no grade C AL group. AAC, SMAC, and IMAC were not independent predictive factors for AL or grade C AL.

AL is one of the most serious complications after RC surgery with intestinal discontinuity.^[10,12]^ The occurrence of AL prolonged the hospitalization, increased the total cost and resulted in mental stress.^[14,22]^ Postoperative mortality and long-term prognosis could be affected as well.^[17]^ Therefore, it was necessary to find out the factors that result in AL after RC surgery, and to help surgeons avoid such adverse events.

Previous studies reported many factors which were associated with AL. These factors included obesity,^[23,24]^ T2DM,^[25]^ tumor location,^[26]^ tumor size,^[27]^ and neoadjuvant therapy.^[28]^ In this study, similar factors were found such as males and lower rectal cancer, furthermore, lower rectal cancer was an independent factor for AL. It might be the reason that lower RC might have poorer blood supply, thus resulting in AL.^[26,29]^ For males, the pelvic cavity was comparatively narrower than females, which increased the difficulty of RC surgery, thus increasing the higher risk of AL.^[30,31]^

Calcification was widely reported in previous studies as an independent factor for AL in esophageal cancer.^[32–34]^ Likewise, calcification was an independent risk factor for gastric cancer patients after gastrectomy.^[35]^ However, it remained unclear whether calcification had similar influence on CRC patients.

There were some differences in the results of previous studies on the relationship between abdominal aortic calcification and anastomotic leakage after laparoscopic colorectal cancer surgery. Morita S et al reported that calcification of the abdominal aorta might be a predictor of AL after laparoscopic CRC surgery.^[36]^ Lee SY et al included 583 RC patients and found that aortoiliac calcification might be considered as a risk factor for grade C AL after RC surgery.^[37]^ Similarly, Shen Z et al reported the predictive roles of calcification.^[38]^ However, Knight KA et al suggested that aortic calcification was not associated with AL.^[39]^ Therefore, it was necessary to analyze the accurate effect of calcification on AL after RC surgery.

Adequate blood perfusion and lower tension were important to the anastomosis healing. As was reported that the mechanism of calcification on AL was that insufficient perfusion of artery due to calcification caused the delayed healing and AL,^[35,36,40]^ especially in end of small artery. In this study, AAC was associated with higher amount of AL and grade C AL, however, SMAC and IMAC were negative indicators. The mechanism was unclear why calcification in larger arteries had higher rates of AL than smaller arteries. It might be related to the difference in baseline information. Further studies are needed in the following experiments.

To our knowledge, the current study has analyzed the relationship between aortic calcification and AL after RC surgery with the largest amount of data, therefore, the results might be more accurate; furthermore, this is the first study to analyze the difference of AL in SMAC and IMAC, however, no significant difference was found; moreover, multivariate logistic regression to identify the predictive roles of AAC, SMAC, and IMAC.

This study had some limitations. First, this was a single center retrospective study, selection bias was hard to avoid because of retrospectively collecting patients’ information from one clinical center; second, the long-term roles of AL or AAC on the outcomes of RC patients were not followed up. Therefore, multi-center with large-sample size studies are needed in the future to identify the accurate relationship between calcification and AL after RC surgery.

In conclusion, AAC was associated with higher amount of AL and grade C AL, however, AAC was not an independent predictive factor for AL or grade C AL.

## Acknowledgments

We acknowledge all the authors whose publications are referred in our article.

## Author contributions

**Conceptualization:** Dong Peng, Dong Yang.

**Data curation:** Yu-Hang Diao, Yang Liu, Dong Peng, Dong Yang.

**Formal analysis:** Yang Liu, Dong Peng.

**Project administration:** Yang Liu, Dong Peng.

**Software:** Yu-Hang Diao.

**Supervision:** Yang Liu, Dong Peng, Dong Yang.

**Validation:** Jian Chen, Yang Liu, Dong Peng, Dong Yang.

**Visualization:** Dong Peng.

**Writing – original draft:** Yu-Hang Diao, Dong Yang.

**Writing – review & editing:** Yu-Hang Diao, Jian Chen, Dong Yang.
